# Longitudinal Study of the Distribution of Antimicrobial-Resistant *Campylobacter* Isolates from an Integrated Broiler Chicken Operation

**DOI:** 10.3390/ani11020246

**Published:** 2021-01-20

**Authors:** Bo-Ram Kwon, Bai Wei, Se-Yeoun Cha, Ke Shang, Jun-Feng Zhang, Min Kang, Hyung-Kwan Jang

**Affiliations:** 1 Department of Veterinary Infectious Diseases and Avian Diseases, College of Veterinary Medicine and Center for Poultry Diseases Control, Jeonbuk National University, Iksan 54596, Korea; aniscikwon@gmail.com (B.-R.K.); weibai116@hotmail.com (B.W.); kshmnk@hanmail.net (S.-Y.C.); shangke0624@gmail.com (K.S.); jfzhang018@gmail.com (J.-F.Z.); 2 Bio Disease Control (BIOD) Co., Ltd., Iksan 54596, Korea

**Keywords:** *Campylobacter*, whole-chicken production chain, antimicrobial resistance, longitudinal study, PFGE, genetic diversity

## Abstract

**Simple Summary:**

Investigation of *Campylobacter* prevalence throughout the entire chicken production process from farms to retail meat is still limited. In this study, we examined the prevalence and antimicrobial susceptibility of *Campylobacter* in 10 production lines from one of the largest integrated poultry production companies in Korea. The prevalence of *Campylobacter* in breeder farm, hatchery, broiler farm, slaughterhouse, and retail meat products was 50.0%, 0%, 3.3%, 13.4%, and 68.4%, respectively. Resistance to fluoroquinolones was the most frequently observed, and 16 isolates from breeder farm were resistant to both azithromycin and ciprofloxacin. Diverse pulsed-field gel electrophoresis genotypes were presented with discontinuous patterns along the whole production chain. Thirty percent of *Campylobacter*-free flocks became positive after slaughtering. An identical genotype was simultaneously detected from both breeder farm and retail meat, even from different production lines. This study reveals that antimicrobial-resistant *Campylobacter* contamination can occur at all stages of the chicken supply chain. In particular, the breeder farm and slaughterhouse should be the main control points, as they are the potential stages at which antimicrobial-resistant *Campylobacter* could spread to retail meat products by horizontal transmission.

**Abstract:**

The aim of this study was to analyze the prevalence, antimicrobial resistance, and genetic diversity of *Campylobacter* isolates that were obtained from whole chicken production stages in Korea. A total of 1348 samples were collected from 10 production lines. The prevalence of *Campylobacter* in breeder farm, broiler farm, slaughterhouse, and retail meat products was 50.0%, 3.3%, 13.4%, and 68.4%, respectively, and *Campylobacter* was not detected at the hatchery stage. Resistance to quinolones/fluoroquinolones was the most prevalent at all stages. Among the multidrug-resistant isolates, 16 isolates (19.8%) from breeder farm were resistant to both azithromycin and ciprofloxacin. A total of 182 isolates were subdivided into 82 pulsed-field gel electrophoresis (PFGE) genotypes with 100% similarity. Diverse genotypes were presented with discontinuous patterns along the whole production chain. Thirty percent of *Campylobacter*-free flocks became positive after slaughtering. An identical genotype was simultaneously detected from both breeder farm and retail meat, even from different production lines. This study reveals that antimicrobial-resistant *Campylobacter* contamination can occur at all stages of the chicken supply chain. In particular, the breeder farm and slaughterhouse should be the main control points, as they are the potential stages at which antimicrobial-resistant *Campylobacter* could spread to retail meat products by horizontal transmission.

## 1. Introduction

*Campylobacter* spp. are a leading cause of food-borne diarrheal illnesses globally, and *Campylobacter* infection is among the most frequently reported causes of gastroenteritis in humans worldwide [1]. Poultry and poultry products, particularly contaminated chicken products, are considered to be major sources of human infection [2]. *Campylobacter* can be isolated at all stages of the chicken supply chain from farms to retail meat products [3]. *Campylobacter* usually colonizes from the third week of age after the beginning of the rearing period and, once colonized, *Campylobacter* will rapidly reach high numbers in flocks and the farm environment [4]. Many studies have found that *Campylobacter* is rarely detected in day-old chicks, possibly due to the protection that is offered by maternal antibodies [2]. According to a previous study, vertical transmission from parent flocks to their progeny still remains unknown [5]; meanwhile, several suspected horizontal transmission sources or vectors, including the poultry house environment, small animals on the farm, flies, and rodents, have been identified as major factors of flock colonization [4]. Various contamination factors specifically exist in slaughterhouses; for example, direct contact between carcasses can frequently induce cross-contamination during defeathering and evisceration, and contact with common surfaces, such as rubber fingers, conveyor belts, and cutting tables, is also a main reason for cross-contamination and the presentation of various colonies of *Campylobacter* [6,7]. Some isolates seem to survive in the slaughter equipment and during processing [7]. The persistence of *Campylobacter* in the equipment may lead to the contamination of *Campylobacter*-negative flocks that are slaughtered after *Campylobacter*-positive flocks [8]. Numerous studies concluded that the most effective measures should aim at reducing the prevalence of *Campylobacter*-positive flocks and the level of contamination of *Campylobacter* on broiler carcasses [9].

When *Campylobacter* infection requires antimicrobial treatment, macrolides and fluoroquinolones are normally considered to be first and second antimicrobials of choice, respectively [10]. However, the recent emergence of resistance to these antimicrobial groups and multidrug-resistant *Campylobacter* isolates has been observed within the food supply chain [3]. The use of enrofloxacin in poultry was banned in the US in 2005 because increased levels of fluoroquinolone resistance have raised public health concern. In Korea, the use of antimicrobial agents as growth promoters was withdrawn in 2011, but antimicrobial agents, including quinolone, macrolides, tetracyclines, and penicillin, are still widely used in the conventional chicken industry for treating diseases [11]. Despite the policy against antimicrobial usage, persistent use of antimicrobial agents may induce the development of resistance and affect other properties, such as the ability to colonize an animal host or persist in the farm or food processing environment [12].

Most studies monitoring the prevalence and antimicrobial resistance of *Campylobacter* have focused on slaughterhouse and retail meat [13,14,15,16]. However, investigation covering the whole chicken production stage from farms to retail meat are still limited [17]. Tracing the distribution of *Campylobacter* longitudinally in whole chicken production stages would help to identify the relatedness of transmission to subsequent stages and determine the mode of transmission between the vertical and horizontal routes. We examined the most prevalent contamination spot and antimicrobial susceptibility of *Campylobacter* in 10 production lines from integrated chicken operation. Given that resistant *Campylobacter* strains could be directly transmitted to the people who had direct contact with the contaminated breeder chicken [18], we included breeder farm as the beginning of production stage and observed the prevalence of antimicrobial resistant *Campylobacter*, especially those that are resistant to fluoroquinolone and/or azithromycin; both of which are used widely in human. In addition, pulsed-field gel electrophoresis (PFGE), which is widely regarded as the gold standard for tracing outbreaks [7], was performed for molecular typing of *Campylobacter* isolates in order to clarify the transmission routes and epidemiological relationships among isolates of the same species.

## 2. Materials and Methods

### 2.1. Description of Production Company and Farms 

From August 2015 to August 2016, 10 chicken production lines (lines 1 to 10), which belonged to one of the largest integrated poultry production company in South Korea, were chronologically investigated from breeder farms to retail meat products. The breeder farms were located in various provinces. The size of breeder farm flocks varied from 16,000 to 50,000 chickens. Every broiler hatching egg produced by these farms was transported to hatchery assigned to same integrated company. Newly hatched chicks were then again transported to, and reared at, broiler farms, which contained an average of 70,000–100,000 broilers and three to five separate flocks, until their slaughter age of 30 days. Finally, chickens from all broiler farms in this study were gathered and slaughtered in one processing plant. All of the breeder and broiler farms in this study used ampicillin, florfenicol, and tetracycline for disease treatment. 

### 2.2. Sampling and Isolation

All of the animals used were commercially raised and reared in conventional chicken farms under the supervision of the local veterinary authorities; in particular, sampling was performed in accordance with the relevant guidelines (Guide for the Care and Use of Laboratory Animals 2014, Korean Ministry of Food and Drug Safety) and regulations (Korean Council on Animal Care and Korean Animal Protection Law, 2015; Article 23) for experiments with livestock animals in farm. No chickens were killed for this study, and sampling was carried by a veterinarian according to the standard protocols and with prior consent of the farmer/manager of the facilities. Furthermore, written informed consent was obtained from the owners for the participation of their animals in this study.

In order to increase the relatedness between samples, the sampling procedure was implemented in an orderly manner from breeder farms to retail meat, and sampling was limited to one cycle—from breeder farm to production as retail meat; furthermore, the samples were acquired as evenly as possible in order to avoid oversampling at a specific time or region. From 10 chicken production lines, a total of 1348 samples from all stages, such as breeder farm, hatchery, broiler farm, slaughterhouse, and retail meat, were collected (Supplementary Table S1). Cloacal swab samples (n = 110) from 28–65-week-old chickens and litter samples (n = 66) were collected from breeder farms (n = 176). In hatcheries, cloacal swab samples were collected from newly hatched chicks (n = 165). All of the cloacal swab samples that were collected from breeder farms and hatcheries were separately pooled from five chickens to one. As for broiler farms, two flocks per farm were sampled three times within a 30-day period (chickens at 1–14 d, 15–24 d, and >25 d of age) during one rearing cycle. Broiler farm sampling was repeated during a second, separate production cycle (n = 720). The cloacal swab samples (n = 300) were randomly collected from 25 chickens in the entire area of the flock. In detail, a flock was divided equally into five sectors, and five cloacal samples were obtained from each sector and then pooled into one sample, making five samples in total for a flock. Environmental samples of feed (n = 120), litter (n = 180), and water (n = 120) were uniformly collected from equally divided sectors of the flock, and each sample from the same sector was pooled into one sample (Supplementary Table S1). The samples from slaughterhouse (n = 230) were collected at the beginning of each sampling day during the slaughtering of the first batch of broilers chickens; different production lines were sampled at different sampling dates. From lairage, five cloacal swab samples from five different chicken were collected, which were then pooled into one sample (n = 50). Furthermore, environmental samples in slaughterhouse were collected by aseptically swabbing on the surface of each slaughtering site; they were also pooled into one sample (n = 180). Retail meat samples (n = 57) were collected from the meat that was purchased from retail markets in Jeonbuk province, South Korea. All of the samples were placed into plastic bags and boxes and then transported in a box with ice to the laboratory where they were analyzed immediately.

Pooled cloacal swab samples and environmental swab samples were pre-enriched in Bolton broth (Oxoid Ltd., Basingstoke, UK) that was supplemented with cefoperazone, vancomycin, trimethoprim, and cycloheximide (Oxoid). Fresh samples (1 g (or mL)) of feed, litter, and water were separately mixed with 9 mL (1:9 dilution) of Bolton broth. Subsequently, these samples were incubated in a microaerophilic environment of 10% CO_2_, 5% O_2_, and 85% N_2_ at 42 °C for 48 h for enrichment. Each retail meat sample was aseptically rinsed with 100 mL of buffered peptone water (Difco, Sparks, MD, USA) in sterile plastic bags [19]. From rinsed meat, 10 mL of rinse solution was added to 10 mL of 2× Bolton broth. Next, the samples were incubated, as above. After enrichment for 48 h, a loop full of each sample was streaked onto a plate of modified charcoal cefoperazone deoxycholate agar (mCCDA) that was prepared with *Campylobacter* blood-free selective agar base (Oxoid) supplemented with a CCDA selective supplement containing cefoperazone and amphotericin (Oxoid). After incubation, the plates were examined for typical colonies, which are generally small, gray, shiny, and drop-like in shape. At least three presumptive *Campylobacter* colonies from each selective agar plate were further cultured on 5% sheep blood agar plates (Komed, Seongnam, South Korea) microaerobically at 42 °C for 48 h. Presumptive *Campylobacter* isolates were confirmed by polymerase chain reaction (PCR) assay, as described previously [20]. After identifying each isolate, *Campylobacter* isolates were stored in brain heart infusion broth (Oxoid) with 20% glycerol at −70 °C.

### 2.3. Antimicrobial Susceptibility Testing

The susceptibility of all *Campylobacter* isolates to 11 antimicrobial agents was determined by agar dilution method and using Sensititre susceptibility plates (TREK Diagnostic Systems, Incheon, Korea). The standard agar dilution method, as described by the Clinical Laboratory Standards Institute [21], was followed in order to confirm the susceptibility to two antimicrobial agents, namely enrofloxacin (ENR; Daesung Microbiological, Uiwang, Korea) and ampicillin (AMP; Sigma-Aldrich, St. Louis, MO, USA). Mueller–Hinton agar (Oxoid) plates supplemented with 5% lysed sheep blood (Oxoid) and antimicrobial agents at concentrations of 0.125–128 μg/mL for ENR and 8–128 μg/mL for AMP in two-fold serial dilutions were used. Plates were inoculated with 1-mm-diameter inoculating pins and incubated at 42 °C for 24 h under microaerobic conditions. The rest of the nine antimicrobial agents were tested by Sensititre susceptibility plates containing azithromycin (AZM; 0.015–64 μg/mL), erythromycin (ERY; 0.03–64 μg/mL), telithromycin (TEL; 0.015–8 μg/mL), nalidixic acid (NAL; 4–64 μg/mL), ciprofloxacin (CIP; 0.015–64 μg/mL), clindamycin (CLI; 0.03–16 μg/mL), gentamicin (GEN; 0.12–32 μg/mL), florfenicol (FFN; 0.03–64 μg/mL), and tetracycline (TET; 0.06–64 μg/mL). The plates were incubated under microaerobic conditions at 42 °C for 24 h. The results were evaluated according to the interpretation criteria of the National Antimicrobial Resistance Monitoring System [22]. We used the breakpoints for *Enterobacteriaceae* from the Clinical and Laboratory Standards Institute criteria, as no enrofloxacin and ampicillin breakpoints are available for *Campylobacter* [23]. *Campylobacter jejuni* ATCC 33560 was used as a quality control isolate. Multidrug resistant (MDR) isolates were those with resistance to two or more classes of antimicrobials.

### 2.4. Pulsed-Field Gel Electrophoresis (PFGE)

The isolates of *C. coli* and *C. jejuni* were genotyped while using PFGE according to protocols from the Centers for Disease Control and Prevention available on PulseNet. Genomic DNA (extraction using 1% sodium dodecylsulfate and 1-mg/mL proteinase K, Biosesang, Seoul, Korea) of *Campylobacter* isolates was digested with *Sma*I (Thermo Fisher Scientific, Inchon, Korea), and *Xba*I-digested DNA from *Salmonella* Braenderup H9812 was used as the standard size. The PFGE results were analyzed using BioNumerics (version 6.6 for Windows, Kortrijk, Belgium). Dice coefficients were calculated based on a pairwise comparison of the PFGE types of the isolates. The isolates were defined as closely related based on molecular typing when their PFGE patterns had dice coefficients with 100% similarity level. Dice coefficients, with an optimization of 2.0% and a band position tolerance of 1.5%, were applied.

### 2.5. Statistical Analysis

The prevalence of *Campylobacter* spp. between different production stages was compared with the chi-square test. The statistical significance of the differences in resistance to all antimicrobials between *Campylobacter* spp. was also tested while using chi-square test. Differences were considered to be statistically significant at *p* values less than 0.05.

## 3. Results

### 3.1. Distribution of Campylobacter spp. along the Chicken Production Chain

**Figure 1:**
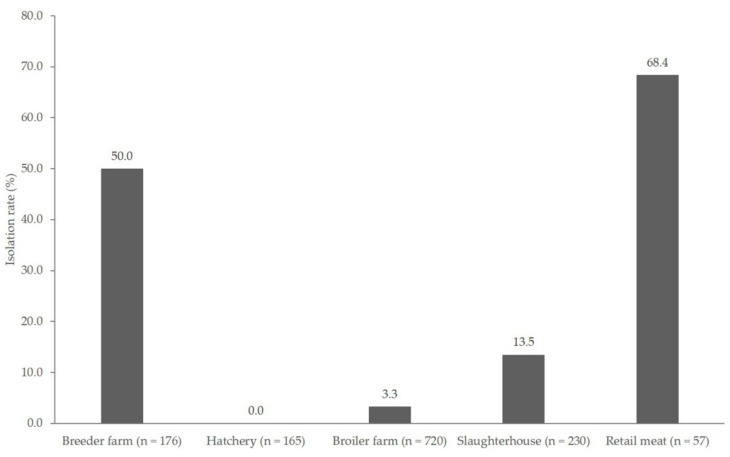
Prevalence of *Campylobacter* isolated from the chicken production chain. (n = total number of samples from each production stage).

The prevalence of *Campylobacter* in breeder, broiler farm, slaughterhouse, and retail meat products was 50.0% (88/176), 3.3% (24/720), 13.5% (31/230), and 68.4% (39/57), respectively (Figure 1), which indicated the highest prevalence in retail meat products (*p* < 0.05). *Campylobacter* was not detected in samples that were acquired from the hatchery stage. The distribution of *Campylobacter* species from the chicken production stage is shown in Table 1. Overall, 182 isolates (13.5%) out of 1348 samples were positive for *Campylobacter*, either *C. coli* (80 isolates, 44%) or *C. jejuni* (102 isolates, 56%). Except in breeder farms and retail meat products, *C. jejuni* was more prevalent than *C. coli* at all other stages. Each production line showed various distribution patterns of *Campylobacter* isolates. Lines 2 and 8 were positive for *Campylobacter* at the breeder farm and retail meat product stages, but not at other stages. Regarding lines 5, 6, and 7, *Campylobacter* spp. were isolated from every stage of the chicken supply chain, except at the hatchery stage.

### 3.2. Antimicrobial Susceptibility

Table 2 presents the results of antimicrobial susceptibility testing performed on the 182 isolates. Resistance to CIP and ENR was the most common (170/182, 93.4%), followed by resistance to NAL (161/182, 88.5%), AMP (133/182, 73.1%), and TET (103/182, 56.6%). Resistance to CLI, GEN, and FFN was only found in 1.6%, 4.4%, and 0.5% samples, respectively. *Campylobacter* resistance to macrolides, such as AZM and ERY, was only noted in isolates that were derived from breeder farms, with resistance rates of 9.9% and 8.8%, respectively. All of the *C. coli* isolates were resistant to at least one antimicrobial tested in this study. The resistance rate for antimicrobials was statistically (*p* < 0.05) higher in *C. coli* than in *C. jejuni* for CIP, ENR, and TET.

Isolates that were resistant to more than two antimicrobial classes were defined as MDR isolates; 57.5% (46/80) of *C. coli* and in 34.3% (35/102) of *C. jejuni* were identified as MDR isolates (Table 3). The most common multidrug resistance pattern in *Campylobacter* spp. was the resistance to quinolones/fluoroquinolones (NAL, CIP, ENR), tetracyclines (TET), and penicillin (AMP). This pattern was observed at all stages of the chicken supply chain. Furthermore, 19.8% (16/81) of MDR isolates were resistant to both AZM and CIP, and they were only detected in samples from breeder farms.

### 3.3. Pulsed-Field Gel Electrophoresis Profiles

After analyzing the PFGE results, the 182 isolates were subdivided into 86 PFGE types with 100% similarity (Table 4). Two predominant types (types 6 and 10) of *C. coli* were associated with six isolates and three predominant types (types 17, 19, and 20) of *C. jejuni* were with nine, 10, and eight isolates, respectively. Most types of *C. coli* (24, 54.5%) and *C. jejuni* (24, 57.1%) were shared with one isolate. There was genotype diversity of the isolates for both *C. coli* and *C. jejuni* in the poultry production chain, with the highest diversity being detected at the breeder stage. The breeder farms carried a large variety of PFGE types, with 30 and 24 types of *C. coli* (Supplementary Figure S1) and *C. jejuni*, respectively (Supplementary Figure S2). The cross-contamination of *C. coli* and *C. jejuni* isolates was common among breeder farms of different production lines. Herein, PFGE type 27 of *C. coli* and PFGE type 9 of *C. jejuni* in a breeder farm were simultaneously found in production lines 4 and 8, which indicated a high frequency of cross-contamination between the two production lines. Moreover, PFGE type 27 of *C. coli* was found in three different production lines (lines 3, 4, and 8), and PFGE types 8 and 21 were found in two different production lines (lines 6 and 7 and lines 6 and 8, respectively). PFGE types 9, 19, and 36 of *C. jejuni* were found in two different production lines, which are lines 4 and 8, lines 1 and 3, and lines 1 and 2, respectively.

The same genotype (type 11 of *C. jejuni*) was simultaneously detected from both breeder farm and retail meat, even from different production lines. PFGE type 19 of *C. jejuni* was found in four different production lines (lines 1, 3, 4, and 5), providing the evidence of contamination across the farm stage, including at the breeder and broiler farm stages. Furthermore, serious cross-contamination between different production lines was found at the slaughterhouse stage. PFGE types 16 and 18 of *C. jejuni* were first found in lines 6 and 9 in slaughterhouses and they were later recovered from retail chicken meat in line 2 and lines 6 and 8, respectively. Some of the new PFGE types (*C. coli* from lines 1, 2, 3, 5, 7, and 9; *C. jejuni* from lines 2, 3, 6, 7, 8, and 10) were recovered from retail meat products but were not detected in previous stages of the same production line. Only one PFGE type (type 19 of *C. jejuni*) continuously existed from breeder farm to slaughterhouse, even in different lines, but the rest of the types did not persist across different stages until the final product. Some PFGE types, such as types 8, 11, 16, 18, and 36 of *C. jejuni*, were sparsely detected from different production lines and stages. Fifteen out of 44 types of *C. coli* and 21 types out of 42 types of *C. jejuni* were considered to be non-MDR isolates. Twelve PFGE types out of 86 were MDR isolates, including those non-sensitive to azithromycin and ciprofloxacin, as identified using human therapeutic treatment.

## 4. Discussion

*Campylobacter* is the most common gastroenteritis-causing pathogen worldwide. Foodborne transmission accounts for most cases of *Campylobacter* infection, and up to 80% of *Campylobacter* infections can be attributed to the consumption of poultry, particularly the consumption of contaminated chicken meat [24]. This study shows that monitoring the distribution of antimicrobial resistant *Campylobacter* and its resistance patterns and tracing the route of transmission from comprehensive longitudinal sampling in the whole production stages are important for better understanding the occurrence resistant *Campylobacter* contamination.

A previous study on *Campylobacter* emergence suggested that *Campylobacter* contamination is due to vertical and horizontal transfer in broiler farms [4]. In the present study, all of the production stages, except hatchery, were contaminated with *Campylobacter* (Figure 1). The finding of a hatchery being *Campylobacter*-negative, despite a *Campylobacter*-positive parent flock, indicates that vertical transmission is not a major infectious route as it was in previous studies [5,25]. Furthermore, horizontal transmission comes across as a major potential source of flock infection via feed, litter, water, footwear, and chicken sheds [4]. 

The implementation of strict biosecurity practices was considered to be effective method to prevent or delay *Campylobacter* colonization in broiler chickens during the rearing period. In addition, low prevalence of *Campylobacter* isolates from broiler farms in this study could be due to a short rearing time of about 30 days before slaughter [26]. This result was consistent with the report that identified slaughter age as a risk factor for *Campylobacter* colonization in broiler chickens and suggested that reducing the rearing period of broiler chicken would decrease the prevalence of *Campylobacter* [27]. However, when compared with the low prevalence of *Campylobacter* in broiler farms and slaughterhouses, the isolation rate rapidly increased in retail meat samples in this study. These results were in accordance with the finding that suggested the possibility of contamination during slaughter [7,9,28]. Therefore, *Campylobacter* control in poultry faces many hurdles that need to be overcome and probably several strategies will have to be combined in order to achieve this goal. Although the best way to reduce *Campylobacter* contamination in chicken carcasses is to prevent colonization in the broiler house, an effective, suitable, and reliable strategy to eradicate this foodborne pathogen should focus not only on rearing farms, but also on the subsequent stages [29].

Most *Campylobacter* isolates (175/182) were resistant to at least one antimicrobial agent. Notably, extremely high resistance to nalidixic acid (88.5%), ciprofloxacin (93.4%), and enrofloxacin (93.4%) were found in this study, which is a finding that is consistent with previous studies [13,19,30]. In addition, 44.5% (81/182) of isolates showed multidrug resistance, and 16 isolates (16/81, 19.8%) were resistant to both azithromycin and ciprofloxacin. Extremely high resistance to fluoroquinolones and a steady increase in macrolide resistance would pose a serious public health threat of the transmission of such resistant *Campylobacter* through the chicken production stages [3]. Contrary to the high resistance to fluoroquinolones (>90%), tetracycline (56.6%), and ampicillin (73.1%), low resistance to gentamicin (4.4%) and florfenicol (0.5%) was identified in this study, which is consistent with the findings of a previous study [31]. Although these antimicrobials (gentamicin and chloramphenicol) are not the routine choice of treatment for human *Campylobacter* infection, increasing the resistance to the first-line antimicrobials and the decline in newly developed antimicrobials necessitated the monitoring of these alternative antimicrobial agents; this is because monitoring antimicrobial resistance is crucial in establishing the prevention and control measures in order to limit the dissemination of the resistant isolates. Thus, enhanced monitoring of *Campylobacter* resistance to these antimicrobials is required in order to better prevent infections that are caused by resistant pathogens and protect public health.

In general, *C. jejuni* was reported as the predominant *Campylobacter* species in poultry. However, our results showed a similar prevalence of *C. coli* and *C. jejuni*. Similar results, showing that the prevalence of *C*. *coli* was similar to that of *C. jejuni* or that *C*. *coli* showed an even higher prevalence than *C. jejuni* in poultry, have been reported in China, Thailand, and Reunion Island, among other places [17,32,33]. In addition, *C*. *coli* always showed higher antimicrobial resistance than *C. jejuni* and, accordingly, the choice of disinfectants and antimicrobials used in farms could be targeted at certain *Campylobacter* populations [32,34,35]. This study shows that *C. coli* demonstrated higher rates of antimicrobial resistance than *C. jejuni* in accordance with previous studies [32,34,35]. Our results suggest that the use of antimicrobial agents, such as ampicillin, florfenicol, and tetracycline, in farms may lead to favorably selected antimicrobial resistant *C. coli* being higher in prevalence than *C. jejuni*. It poses a potential public health threat and, thus, should be monitored in high priority in order to control the widespread of *C. coli*.

In this study, the genetic diversity among *Campylobacter* isolates and the presence of *Campylobacter* isolates along the chicken meat supply chain were evaluated. In contrast to other studies, the discontinuous appearance of *Campylobacter* and the diversity of PFGE types of isolates were mostly present along the entire chicken production process [7,36]. This result suggests that various contamination sources, such as wild animals, insects, farm staff, transport vehicles, and slaughtering environment, and equipment with *Campylobacter*-positive flocks, exacerbate the risk of bringing new resistant isolates into the chicken production stages [4]. In addition, genetic instability has been reported in *Campylobacter* isolates that are highly sensitive to environmental stress both in farms and slaughterhouse [37,38]. Furthermore, we have to acknowledge the limitations that are associated with our small sample sizes for slaughterhouse and retail meat, particularly when compared to the whole flock, which has about 15,000–20,000 broiler chickens; however, a larger number of samples was acquired for several sampling sites [39,40]. 

During the processing of poultry carcasses in slaughterhouse, cross-contamination between production lines seems to be relatively frequent. We found that PFGE type 16 of *C. jejuni* isolates from line 6 in the slaughterhouse was re-isolated from retail meat of line 2. Furthermore, PFGE type 18 of *C. jejuni* isolates from line 9 in the slaughterhouse was re-isolated from retail meat of lines 6 and 8. In addition, a *Campylobacter*-free flock could become positive after processing in the slaughterhouse. From our results, retail meat from 30% (3/10) flocks became *Campylobacter*-positive, even when these flocks (lines 2, 8, and 10) were negative at earlier stages. The primary source of contamination of *Campylobacter* for these *Campylobacter*-free flocks may be the *Campylobacter*-positive flocks that were slaughtered on previous days. These results suggested that some strains of *Campylobacter* form biofilms outside the host and may form a film on metal, glass, or rubber surfaces in the slaughterhouse; furthermore, *Campylobacter* can survive in the slaughter environment, even after cleaning with disinfectants [41,42,43]. Some surviving isolates could persist up to three weeks in the slaughterhouse environment, and these colonies could pose a high contamination risk to the following chicken flock [7,44]. These interventions at the slaughterhouse stage are an urgent requirement, as current interventions against *Campylobacter* contamination during poultry slaughter are not implemented in Asia [45].

We also noted the spread of the same genotype (PFGE type 11 of *C. jejuni*) that was isolated from breeder chicken and retail meat from different production lines. This result corroborated a previous study, which reported that the breeder chicken was the reservoir of *Campylobacter* with antimicrobial resistance and that these resistant *Campylobacter* may horizontally or vertically spread to retail meat along the chicken production stages [4,25]; our results are also in agreement with another study, stating that these resistant *Campylobacter* isolates could be directly transmitted to people who come into direct contact with the contaminated breeder chicken [18]. In agreement with previous studies, our results showed that *Campylobacter* isolates from the breeder chicken had higher antimicrobial resistance than those from broiler chicken; furthermore, the breeder chicken also had a higher prevalence of isolates with co-resistance to azithromycin and ciprofloxacin than broiler chicken [46]. With a high possibility to obtain antimicrobial treatment in its long life cycle, breeder chicken could accumulate MDR isolates and would be a persistently infected source for spreading the MDR isolates to the environment and downstream broiler chicken or retail meat via horizontal transmission [25,46]. We also noticed that multiple *Campylobacter* genotypes were shared between different breeder farms (types 8, 21, and 27 of *C. coli* and types 9, 19, and 36 of *C. jejuni*), despite the high biosecurity measures being implemented in breeder chicken farms in South Korea [47]. Moreover, one PFGE type (type 19 of *C. jejuni* from lines 1 and 3) was transmitted from the breeder farm to downstream production stages beyond production lines. The presence of the same genotypes at different production stages and in different lines highlights a common source from the same company that could be shared during the transport of birds, feeding, and veterinary visits, among other ways [4]. Based on these factors, the circulation of specific genotypes in an integrated production system could occur. This result was also supported by the fact that the long life cycle of breeder chicken increased the risk of pathogen exchange by increasing the number of encounters among breeder chicken farms [47]. Therefore, the breeder chicken cannot be excluded from the antimicrobial resistance monitoring program to limit and prevent the spread of resistant *Campylobacter*.

## 5. Conclusions

In conclusion, we found that the significant contamination of antimicrobial-resistant *Campylobacter* was prevalent at all production stages, except at the hatchery stage; moreover, the transmission of *Campylobacter* occurred by multiple routes and it induced a variety of genotypes. To our knowledge, this is the first report on the occurrence of antimicrobial-resistant *Campylobacter* investigated longitudinally from breeder farms to retail meat along the chicken supply chain in Korea. High prevalence of antimicrobial-resistant *Campylobacter* in breeder farms according to the bird age suggests that epidemiological investigations should include breeder farms, which could be a source of transmission of antimicrobial-resistant *Campylobacter*, including the antimicrobials that were used in human treatment, in the chicken supply chain. According to the PFGE results, new types were mainly introduced at farm and slaughterhouse stages with numerous factors that resulted in the accumulation of various genotypes. In particular, the slaughtering process may contaminate *Campylobacter*-negative flocks with various genotypes by the end of the process. These findings indicated that further studies are necessary in order to figure out the contamination factors or routes from rearing farm and slaughterhouse and develop interventions targeting slaughterhouse for improving food safety and public health.

## Figures and Tables

**Table 1:** Distribution of *Campylobacter coli* and *C. jejuni* isolated from different lines along the chicken production chain.

Line	*C. coli* (80/182, 44.0%)							*C. jejuni* (102/182, 56.0%)						
	Breeder n/(%)	Hatchery n/(%)	Broiler n/(%)			SlaughterHouse n/(%)	Retail Meat n/(%)	Breeder n/(%)	Hatchery n/(%)	Broiler n/(%)			SlaughterHouse n/(%)	Retail Meat n/(%)
			1 d–14 d	15 d–24 d	>25 d					1 d–14 d	15 d–24 d	>25 d		
1	4/32 (12.5)	0/20 (0.0)	0/24 (0.0)	0/24 (0.0)	0/24 (0.0)	5/34 14.7) ^c^	3/3 (100.0)	8/32 (25.0)	0/20 (0.0)	0/24 (0.0)	0/24 (0.0)	0/24 (0.0)	0/34 (0.0)	0/3 (0.0)
2	1/16 (6.3)	0/20 (0.0)	0/24 (0.0)	0/24 (0.0)	0/24 (0.0)	0/44 (0.0)	8/14 (57.1)	7/16 (43.8)	0/20 (0.0)	0/24 (0.0)	0/24 (0.0)	0/24 (0.0)	0/44 (0.0)	2/14 (14.3)
3	10/24 (41.7)	0/10 (0.0)	0/24 (0.0)	0/24 (0.0)	0/24 (0.0)	0/34 (0.0)	4/7 (57.1)	10/24 (41.7)	0/10 (0.0)	0/24 (0.0)	0/24 (0.0)	0/24 (0.0)	5/34 (14.7) ^c^	3/7 (42.9)
4	6/16 (37.5)	0/10 (0.0)	0/24 (0.0)	0/24 (0.0)	0/24 (0.0)	0/34 (0.0)	0/3 (0.0)	3/16 (18.8)	0/10 (0.0)	0/24 (0.0)	0/24 (0.0)	0/24 (0.0)	1/34 (2.9) ^c^	0/3 (0.0)
5	4/32 (12.5)	0/20 (0.0)	0/24 (0.0)	0/24 (0.0)	4/24 (16.7) ^a^	2/5 (40.0) ^b^	1/3 (33.3)	8/32 (25.0)	0/20 (0.0)	0/24 (0.0)	0/24 (0.0)	1/24 (4.7) ^a^	0/5 (0.0)	0/3 (0.0)
6	8/16 (50.0)	0/25 (0.0)	0/24 (0.0)	0/24 (0.0)	0/24 (0.0)	0/5 (0.0)	0/6 (0.0)	1/16 (6.3)	0/25 (0.0)	6/24 (25.0) ^a^	0/24 (0.0)	0/24 (0.0)	5/5 (100)	2/6 (33.3)
7	4/24 (16.7)	0/20 (0.0)	0/24 (0.0)	0/24 (0.0)	0/24 (0.0)	0/20 (0.0)	5/9 (55.6)	4/24 (16.7)	0/20 (0.0)	1/24 (4.7) ^b^	0/24 (0.0)	0/24 (0.0)	3/20 (15.0) ^b^	4/9 (44.4)
8	8/16 (50.0)	0/20 (0.0)	0/24 (0.0)	0/24 (0.0)	0/24 (0.0)	0/10 (0.0)	0/6 (0.0)	2/16 (12.5)	0/20 (0.0)	0/24 (0.0)	0/24 (0.0)	0/24 (0.0)	0/10 (0.0)	1/6 (16.7)
9	-	0/10 (0.0)	0/24 (0.0)	0/24 (0.0)	0/24 (0.0)	0/39 (0.0)	3/3 (100.0)	-	0/10 (0.0)	0/24 (0.0)	3/24 (12.5) ^b^	0/24 (0.0)	10/39 (25.6) ^d^	0/3 (0.0)
10	-	0/10 (0.0)	0/24 (0.0)	0/24 (0.0)	0/24 (0.0)	0/5 (0.0)	0/3 (0.0)	-	0/10 (0.0)	4/24 (16.7) ^a^	0/24 (0.0)	5/24 (20.8) ^b^	0/5 (0.0)	3/3 (100.0)
Total	45/176 (25.6)	0/165 (0.0)	0/240 (0.0)	0/240 (0.0)	4/240 (1.7)	7/230 (3.0)	24/57 (42.1)	43/176 (24.4)	0/165 (0.0)	11/240 (4.6)	3/240 (1.3)	6/240 (2.5)	24/230 (10.4)	15/57 (26.3)

^a^ Isolates including cloacal swab and rearing materials (feed, litter, and water). ^b^ Isolates only from cloacal swab. ^c^ Isolates only from environmental sources. ^d^ Isolates including cloacal swab and environmental sources. All positive isolates from breeder farm were isolated from cloacal swab. - Sampling was not included.

**Table 2:** Antimicrobial resistance profiles of *Campylobacter* isolates from the chicken production chain.

Antimicrobial Agent	*Campylobacter* spp.	*C. coli*					*C. jejuni*				
	Total (n = 182)	Breeder (n = 45)	Broiler (n = 4)	Slaughterhouse (n = 7)	Retail Meat (n = 24)	Total (n = 80)	Breeder (n = 43)	Broiler (n = 20)	Slaughterhouse (n = 24)	Retail Meat (n = 15)	Total (n = 102)
Azithromycin	18 (9.9%)	10 (22.2%)	0 (0.0%)	0 (0.0%)	0 (0.0%)	10 (12.5%)	8 (18.6%)	0 (0.0%)	0 (0.0%)	0 (0.0%)	8 (7.8%)
Erythromycin	16 (8.8%)	9 (20.0%)	0 (0.0%)	0 (0.0%)	0 (0.0%)	9 (11.3%)	7 (16.3%)	0 (0.0%)	0 (0.0%)	0 (0.0%)	7 (6.9%)
Telithromycin	11 (6.0%)	7 (15.6%)	0 (0.0%)	0 (0.0%)	0 (0.0%)	7 (8.8%)	4 (9.3%)	0 (0.0%)	0 (0.0%)	0 (0.0%)	4 (3.9%)
Nalidixic acid	161 (88.5%)	43 (95.6%)	4 (100.0%)	5 (71.4%)	19 (79.2%)	71 (88.8%)	43 (100.0%)	16 (80.0%)	16 (66.7%)	15 (100.0%)	90 (88.2%)
Ciprofloxacin	170 (93.4%)	45 (100.0%)	4 (100.0%)	7 (100.0%)	24 (100.0%)	80 (100%)	43 (100.0%)	16 (80.0%)	16 (66.7%)	15 (100.0%)	90 (88.2%)
Enrofloxacin	170 (93.4%)	45 (100.0%)	4 (100.0%)	7 (100.0%)	24 (100.0%)	80 (100%)	43 (100.0%)	16 (80.0%)	16 (66.7%)	15 (100.0%)	90 (88.2%)
Clindamycin	3 (1.6%)	0 (0.0%)	0 (0.0%)	0 (0.0%)	0 (0.0%)	0 (0.0%)	3 (7.0%)	0 (0.0%)	0 (0.0%)	0 (0.0%)	3 (3.0%)
Gentamicin	8 (4.4%)	6 (13.3%)	0 (0.0%)	0 (0.0%)	0 (0.0%)	6 (7.5%)	2 (4.7%)	0 (0.0%)	0 (0.0%)	0 (0.0%)	2 (2.0%)
Florfenicol	1 (0.5%)	0 (0.0%)	0 (0.0%)	0 (0.0%)	0 (0.0%)	0 (0.0%)	1 (2.3%)	0 (0.0%)	0 (0.0%)	0 (0.0%)	1 (1.0%)
Tetracycline	103 (56.6%)	29 (64.4%)	4 (100.0%)	7 (100.0%)	20 (83.3%)	60 (75.0%)	23 (53.5%)	6 (30.0%)	5 (20.8%)	9 (60.0%)	43 (42.2%)
Ampicillin	133 (73.1%)	35 (77.8%)	4 (100.0%)	5 (71.4%)	18 (75.0%)	62 (77.5%)	39 (90.7%)	12 (60.0%)	8 (33.3%)	12 (80.0%)	71 (69.6%)

**Table 3:** Antimicrobial resistance patterns of *Campylobacter coli* and *C. jejuni* isolates from the chicken production chain.

No. of Antimicrobial Agents	Antimicrobial Resistance Pattern	n ^a^ (%)	No. of *C. coli* in Each Stage				No. of *C. jejuni* in Each Stage			
			Breeder	Broiler	Slaughterhouse	Retail Meat	Breeder	Broiler	Slaughterhouse	Retail Meat
	Susceptible	7 (3.8)							7	
1	AMP	5 (2.7)						4	1	
2	CIP+ENR	2 (1.1)	1			1				
3	NAL+CIP+ENR	27 (14.8)	9				4	8	5	1
3	CIP+ENR+AMP	2 (1.1)	1			1				
4	NAL+CIP+ENR+AMP	44 (24.2)	12			2	16	2	7	5
4	NAL+CIP+ENR+TET	14 (7.7)			3	5	1		3	2
4	CIP+ENR+TET+AMP	5 (2.7)			2	3				
5	NAL+CIP+ENR+TET+AMP	54 (29.7)	12	4	1	11	12	6	2	6
5	NAL+CIP+ENR+GEN+AMP	1 (0.5)	1							
6	NAL+CIP+ENR+GEN+TET+AMP	4 (2.2)	3				1			
6	AZM+NAL+CIP+ENR+TET+AMP	1 (0.5)					1			
6	NAL+CIP+ENR+FFN+TET+AMP	1 (0.5)					1			
7	AZM+ERY+NAL+CIP+ENR+TET+AMP	4 (2.2)	1				3			
7	AZM+NAL+CIP+ENR+GEN+TET+AMP	1 (0.5)	1							
8	AZM+ERY+NAL+CIP+ENR+GEN+TET+AMP	2 (1.1)	1				1			
8	AZM+ERY+TEL+NAL+CIP+ENR+TET+AMP	6 (3.3)	6							
8	AZM+ERY+NAL+CIP+ENR+CLI+TET+AMP	2 (1.1)					2			

AZM: Azithromycin, ERY: Erythromycin, TEL: Telithromycin, NAL: Nalidixic acid, CIP: Ciprofloxacin, ENR: Enrofloxacin, CLI: Clindamycin, GEN: Gentamicin, FFN: Florfenicol, TET: Tetracycline, AMP: Ampicillin. ^a^ Number of *Campylobacter* spp.

**Table 4:** Pulsed-field gel electrophoresis type of multi-drug resistant (MDR) *Campylobacter coli* and *C. jejuni* isolates from different lines along the chicken production chain.

Line	MDR *C. coli*				MDR *C. jejuni*			
	Breeder	Broiler	Slaughterhouse	Retail Meat	Breeder	Broiler	Slaughterhouse	Retail Meat
1	12 ^a^,17 ^a^,29 ^a^,38		25 ^c^, 30 ^c^	20, 32	19 ^a^, 27 ^b^, 33 ^a^, 36, 37 ^b^, 41 ^a^			
2	3			6 ^c^, 7, 13 ^a^	2, 5, 10, 12, 36			16 ^a^, 25
3	2, 11 ^a^, 18 ^b^, 27 ^a^, 42 ^a^, 43 ^a^, 44			5, 19 ^b,c^	3 ^a^, 19 ^b,c^, 42		38 ^a^	15, 40 ^a^
4	27 ^a^, 28 ^a^, 37 ^a^				9, 28 ^b^, 32 ^a^		19 ^a^	
5	22 ^b^,26	23, 24	33	34	13, 26 ^b^, 29 ^b,c^, 30 ^a^	19 ^a^		
6	4 ^a^, 8, 14 ^b^, 15 ^b^, 21, 39 ^c^, 40 ^c^				11^a^	35, 36	3 ^a^, 4 ^a^, 16 ^a^	18 ^a^
7	1, 8, 9 ^a^, 41			10 ^c^, 34 ^a^	24 ^a^, 34 ^a^, 39 ^a^	22 ^a^	8 ^a^	6, 11, 21 ^a^, 31
8	16, 21, 27 ^a^, 31 ^b^, 35 ^a^, 36 ^a^				1, 9			18 ^a^
9	-			10	-	22 ^a^, 23 ^a^	14, 18, 20 ^a^	
10	-				-	17 ^a^		7 ^c^, 8 ^a^

^a^ Non-MDR strain. ^b^ Including non-susceptible to azithromycin and/or ciprofloxacin. ^c^ Including non-MDR strain. - Sampling was not included.

## Data Availability

The data presented in this study are available from the corresponding author on reasonable request.

## Supplementary Materials

The following are available online at https://www.mdpi.com/2076-2615/11/2/246/s1, Table S1. Types and number of samples collected throughout the chicken production chain. Figure S1 A dendrogram of *Campylobacter coli Sma*I-PFGE patterns isolated from the chicken production chain and antimicrobial resistance. Isolate names are the following: S: cloacal swab sample; F: feed sample; from 189-C to 189-I: slaughter processing environment; w: age in weeks; d: age in days. Figure S2. A dendrogram of *Campylobacter jejuni Sma*I-PFGE patterns isolated from the chicken production chain and antimicrobial resistance. Isolate names are the following: S: cloacal swab sample; F: feed sample; W: water sample; L: litter sample; from 198-A to 198-H, 196-A, and from 194-E-1 to 194-J-1: slaughter processing environment; w: age in weeks; d: age in days.
