# Supplementary material for: Longitudinal Study of the Distribution of Antimicrobial-Resistant Campylobacter Isolates from an Integrated Broiler Chicken Operation
Source: Animals (Basel). 2021 Jan 20;11(2):246. doi: 10.3390/ani11020246 (PMC7909429; doi:10.3390/ani11020246)
Supplement: Supplementary file 1 [file animals-11-00246-s001.zip › SUPPL. Table 1..pdf]

SUPPL. Table S1. Types and number of samples collected throughout the chicken production chain.

Production Stage	Time Point			Before Pooling		After Pooling	
				No. of Original Sample	Sample Origin (no. of Pooling Samples)	Sample Origin (no. of Pooled Samples)	Total n/(%)
Breeder farm	28 - 65wks			550	Cloaca swab (5)	Cloaca swab (110)	110/176 (62.5%)
				198	Litter (3)	Litter (66)	66/176 (37.5%)
Hatchery	1-day-old			825	Cloaca swab (5)	Cloaca swab (165)	165/165 (100.0%)
Broiler farm	1-day	15-day	25-day	1500	Cloaca swab (5)	Cloaca swab (300)	300/720 (41.7%)
				540	Litter (3)	Litter (180)	180/720 (25.0%)
				240	Feed (2)	Feed (120)	120/720 (16.7%)
				240	Drinking water (2)	Drinking water (120)	120/720 (16.7%)
Slaughterhouse	-			250	Cloaca swab (5)	Cloaca swab (50)	50/230 (21.7%)
				540	Environment (3)	Environment (180)	180/230 (78.3%)
Retail shop	-			-	Meat (-)	-	57 (100.0%)

- Not applicable
