# Supplementary figures and images for: Longitudinal Study of the Distribution of Antimicrobial-Resistant Campylobacter Isolates from an Integrated Broiler Chicken Operation
Source: Animals (Basel). 2021 Jan 20;11(2):246. doi: 10.3390/ani11020246 (PMC7909429; doi:10.3390/ani11020246)

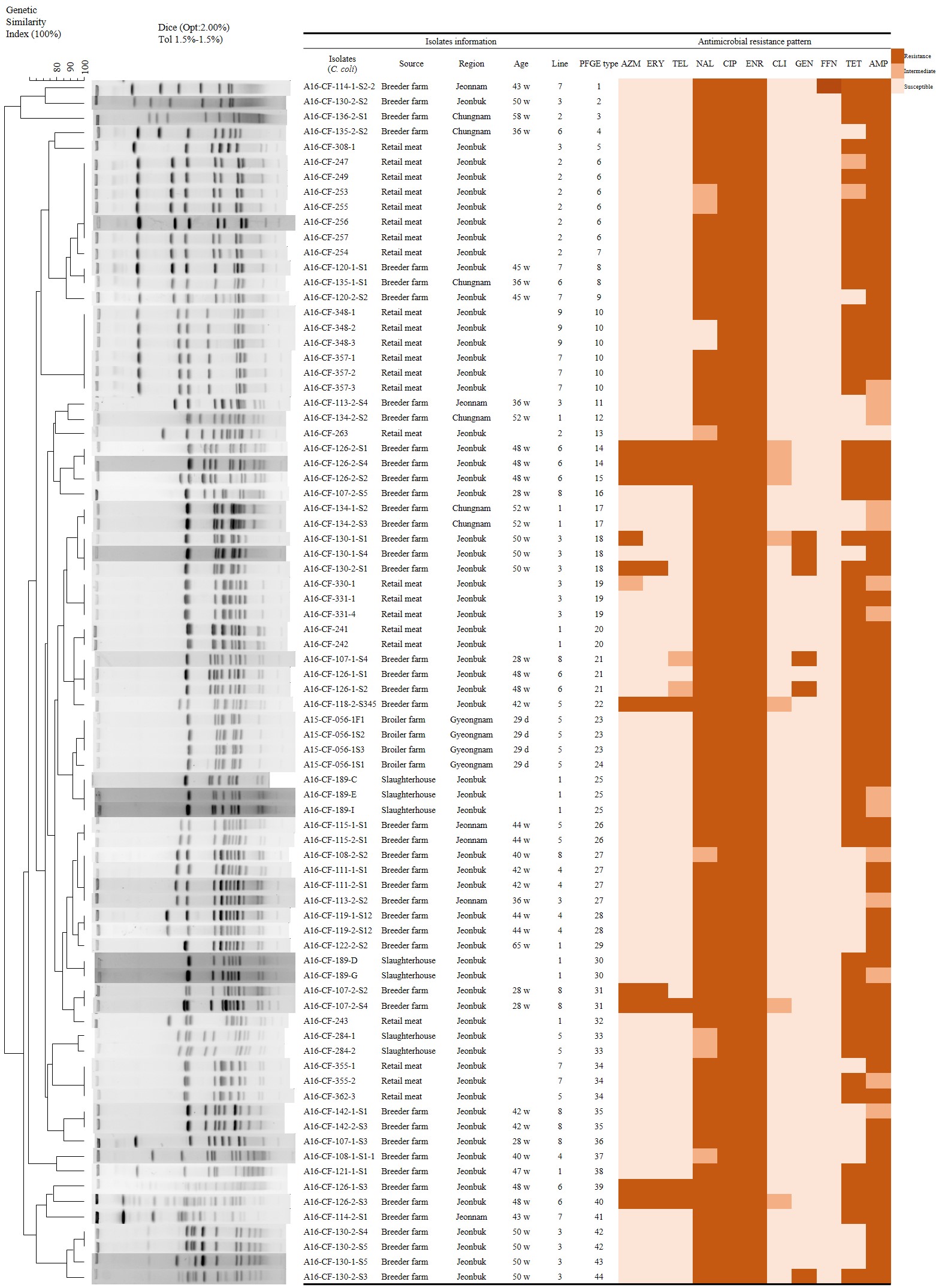

Supplement: Supplementary file 1 [file animals-11-00246-s001.zip › SUPPL. Fig. S1.jpg]

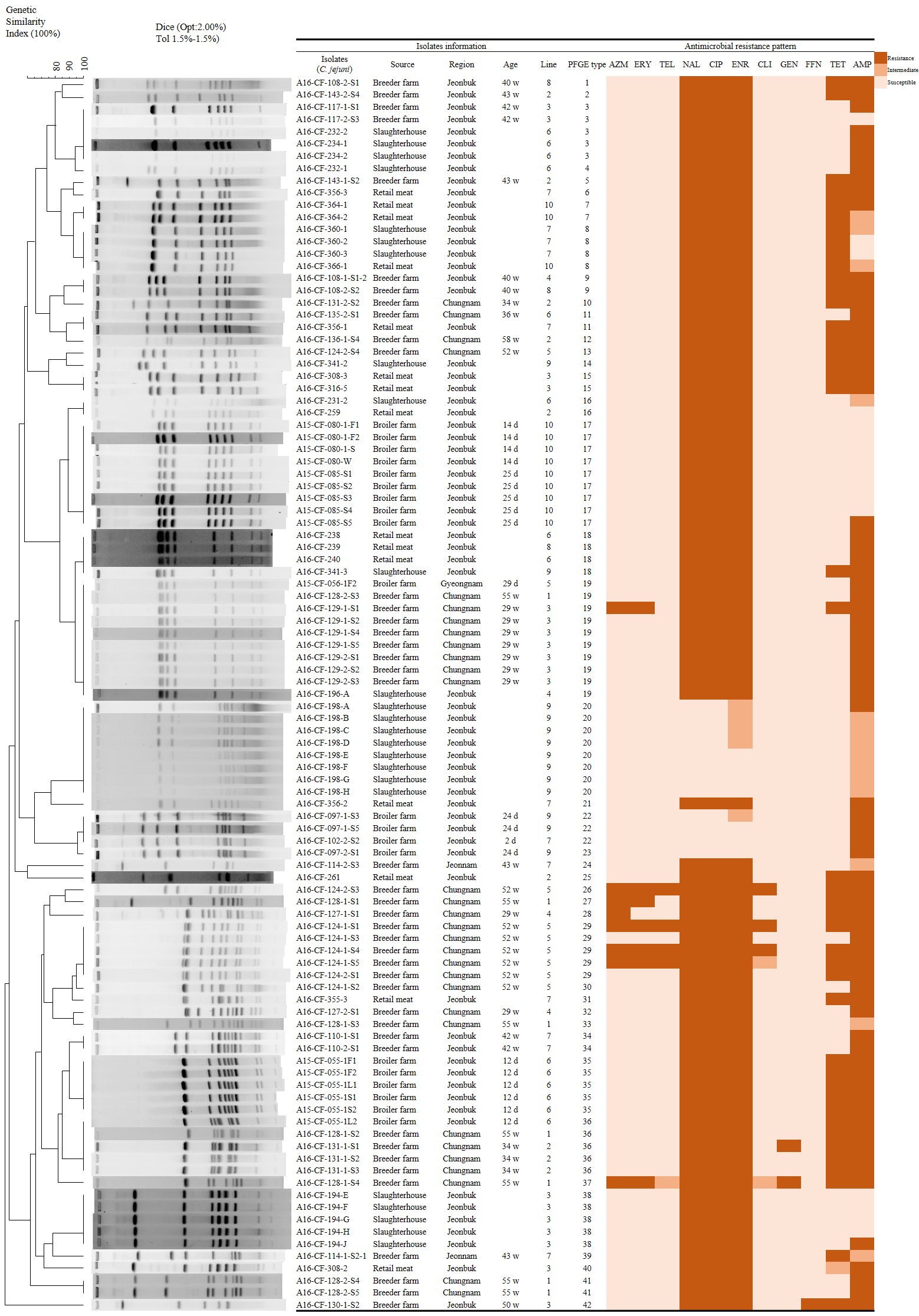

Supplement: Supplementary file 1 [file animals-11-00246-s001.zip › SUPPL. Fig. S2.jpg]
